# The Effects of Certain Processing Technologies on the Cavitation Erosion of Lamellar Graphite Pearlitic Grey Cast Iron

**DOI:** 10.3390/ma18061358

**Published:** 2025-03-19

**Authors:** Eduard Riemschneider, Ion Mitelea, Ilare Bordeașu, Corneliu Marius Crăciunescu, Ion Dragoș Uțu

**Affiliations:** 1 Department of Materials and Fabrication Engineering, Politehnica University Timisoara, Bulevardul Mihai Viteazul nr.1, 300222 Timisoara, Romania; eduard.riemschneider@student.upt.ro (E.R.); ion.mitelea@upt.ro (I.M.); corneliu.craciunescu@upt.ro (C.M.C.); 2 Department of Mechanical Machines, Equipment and Transports, Politehnica University Timisoara, Bulevardul Mihai Viteazul nr.1, 300222 Timisoara, Romania; ilare.bordeasu@upt.ro

**Keywords:** pearlitic grey cast iron, cavitation erosion, alternative technologies

## Abstract

Lamellar graphite pearlitic grey cast irons are frequently used in the manufacturing of components that operate under cavitation erosion conditions. Their poor performance regarding cavitation erosion limits their use in intense cavitation environments. The physical modification of the surface layer offers a flexible and cost-effective way to combat cavitation attacks without altering the core properties. This paper comparatively analyzes the effects of four technological processing methods on the cavitation erosion resistance of grey cast irons. Cavitation erosion tests were conducted on a vibrating device with piezoceramic crystals in accordance with the ASTM G32-2016 standard. Surface hardness tests were carried out using a Vickers hardness tester, while roughness measurements were performed using a Mitutoyo device. The microstructures generated by the applied technologies and the surface wear mechanisms were analyzed using optical microscopy and scanning electron microscopy (SEM). The results indicated that the TIG local surface remelting process provides the most significant improvement in cavitation erosion resistance.

## 1. Introduction

The superior utilization of metallic materials subjected to cavitation erosion during operation requires the adoption of modern technologies that reduce material loss while increasing the durability and performance of products in the automotive, aerospace, and energy industries [1,2,3].

Considering the principles that govern the relationship between the structure and properties of engineering materials, practical methods for influencing these properties through thermal, chemical, and mechanical factors can be suggested. Among these, thermal and thermochemical treatments rely on the dynamics of structural transformations in metals and metal alloys, making them the primary technological tool for directly influencing mechanical and chemical properties [4,5,6,7]. In addition, surface coating techniques—such as cathodic arc plasma deposition [8], mechanical shot peening with a laser beam [9], welding-based surface cladding [10], and TIG (Tungsten inert gas) or laser beam surface remelting [11,12,13]—are effective methods for reducing material losses caused by cavitation erosion. The technological methods selected in this study to improve resistance to cavitation erosion were chosen based on the risk of an ecological hazard and the capabilities of equipment manufacturers operating in cavitational environments. Correlations between cavitation measurements and the mechanical properties of the material, such as hardness, Young’s modulus, tensile strength, yield strength, impact energy (KV), and the product of the fatigue resistance coefficient and the cyclic work-hardening exponent, were only partially successful. Most research studies suggest a strong correlation between hardness values and cavitation resistance.

Gray cast irons with lamellar graphite used in industry are ternary Fe-C-Si alloys that contain Mn, P, and S as unavoidable impurities from the manufacturing process. They are used in the manufacturing of various engineering components that operate in cavitation environments. These components include cylinder liners for internal combustion engines, bodies of devices in hydrostatic actuation systems, double-offset butterfly valves, spiral casings of centrifugal pumps, and hydraulic microturbine chambers. The presence of graphite lamellar in the microstructure of these alloys plays a critical role in cavitation erosion behavior due to the large volume of the metallic matrix intersected by this non-metallic structural constituent.

Numerous processing technologies have been analyzed to prevent the deterioration of these materials due to cavitation erosion.

W.J. Tomlinson and M.G. Talks [14] investigated the effect of 0.2% and 1.0% phosphorus in the chemical composition of lamellar graphite grey cast iron on cavitation erosion behavior in distilled water, NaCl, and NaCl with an inhibitor. Salt in water increased the cavitation erosion rate and the number of pits/cracks on the surface. For 0.2% phosphorus in 3% saltwater, the nominal incubation time was reduced by a factor of 0.2, and the steady-state erosion rate increased sevenfold, with 90% of the increase attributed to corrosion-induced erosion. The presence of a continuous network of brittle phosphide in 1.0% phosphorus grey cast iron had a negligible effect on the erosion rate but reduced the incubation time by a factor of 0.3. The inhibitor completely eliminated corrosion effects up to a 0.25% salt concentration.

Il-Cho Park, Hun-Kee Lee, and Seong-Jong Kim [15] showed that thermochemical nitriding treatment, although it increased surface hardness by approximately 45%, reduced the cavitation erosion resistance. This phenomenon is explained by the formation of a fragile compound layer in the nitrided zone. To minimize material losses, the authors recommended surface modification with a laser beam.

Hattori S. and Kitagawa T. [16] analyzed the cavitation erosion resistance of grey cast iron and non-ferrous alloys compared to unalloyed steels. Their results showed that the cavitation erosion resistance was 1/3 to 1/5 lower for lamellar graphite grey cast iron and 2/3 to 1/3 lower for nodular graphite cast iron compared to unalloyed steel with the same hardness. For aluminum alloys, cavitation erosion resistance was 1/3 to 1/5 lower than that of unalloyed steel, while for copper and titanium alloys, it was approximately equal to that of unalloyed steel.

Wang et al. [17] investigated how the constraints of flat and curved surfaces differently affect the evolution of the cavitation cloud in time and space under submersion conditions. Orthogonal decomposition was used to analyze the flow field characteristics of cavitation along these surface constraints. Additionally, a large eddy simulation (LES) was employed to explore the internal flow dynamics and the progression of the cavitation cloud under different target surface constraints.

The objective of the research presented in this paper is to investigate the cavitation erosion response of several hot processing techniques—stress relief annealing, induction surface hardening, TIG local surface remelting, and plasma nitriding—on the surface integrity of this category of lamellar graphite grey cast iron.

## 2. Materials and Methods

The material studied is EN-GJL-200 grey cast iron, and its chemical profile is presented in Table 1.

Besides iron and carbon as basic elements, the chemical composition also includes permanent accompanying elements (Si, Mn, P, and S). Carbon is the main alloying element, with γ-phase stabilizing (gamagenic) and graphitizing effects, influencing the amount of graphite and pearlite in the base structure. Gamagenic elements (such as C, Cu, Ni, etc.) lower the A3 point and raise the A4 point, expanding the stability range of the γ (austenite) phase while reducing the domain of the α (ferrite) phase. Although increasing the carbon content improves castability, it deteriorates the mechanical properties by increasing the proportion of graphite in the microstructure.

Silicon dissolves by substitution in α-iron, exhibiting a ferritizing effect and a strong graphitizing effect, as it significantly reduces carbon solubility in austenite. Manganese is a γ-phase stabilizing and anti-graphitizing element, phosphorus has a slight graphitizing effect, while sulfur is a strong anti-graphitizing and pearlitizing element (accelerating the decomposition of austenite into pearlite).

**Figure 1:**
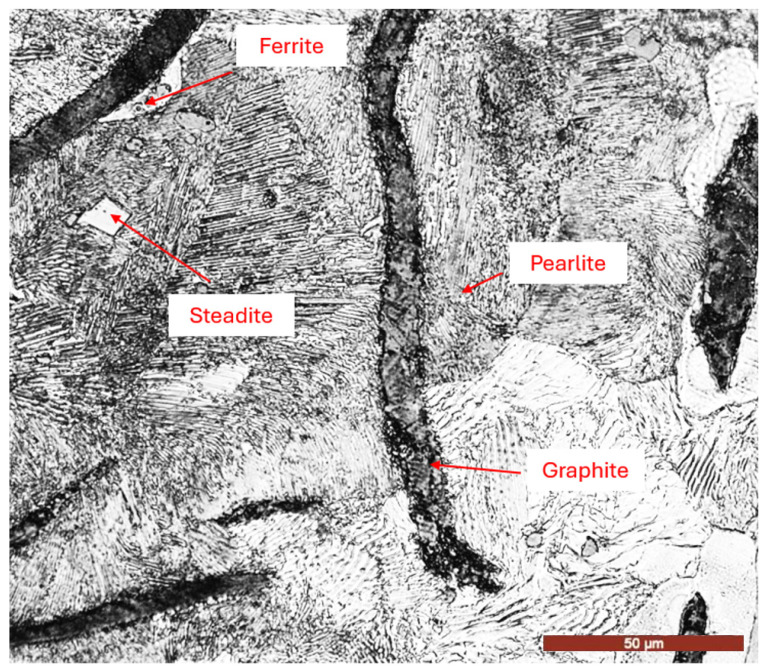
Equilibrium microstructure of EN-GJL-200 grey cast iron.

The SEM microstructural image in Figure 1 depicts the reference material thermally treated by stress-relief annealing. Alongside graphite, the presence of lamellar pearlite with varying degrees of dispersion, rare islands of phosphorous eutectic (steadite), and a very small proportion of ferrite surrounding the graphite precipitates can be observed. This phenomenon can be explained by the fact that the regions of austenite in contact with graphite are carbon-depleted, making them more likely to transform into ferrite during the eutectoid transformation compared to more distant regions.

The predominant role of graphite in the reduced mechanical characteristics of grey cast iron is due to

- The notch effect, which reduces the effective cross-sectional area for bearing mechanical loads.
- The insulation effect, where certain regions of the base material are isolated, leading to uneven and discontinuous stress distribution—particularly when graphite is distributed interdendritically in the form of compact walls or networks.
- The stress concentration effect, where the mechanical stresses can be concentrated up to 10–100 times the average value, especially at the tips of lamellar graphite, causing premature fracture along the graphite lamellae.

For most applications, the most suitable microstructure consists of a pearlitic base matrix and uniform lamellar graphite of type A [14,15].

**Figure 2:**
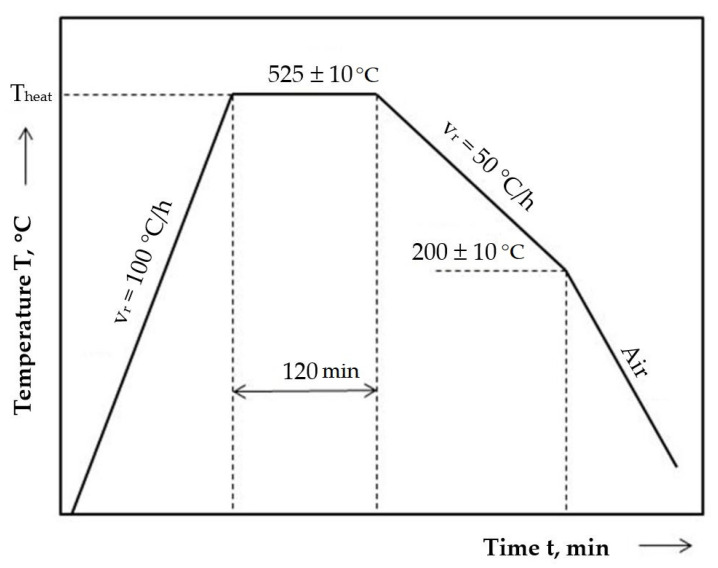
Cycle diagram of stress relief annealing heat treatment.

**Figure 3:**
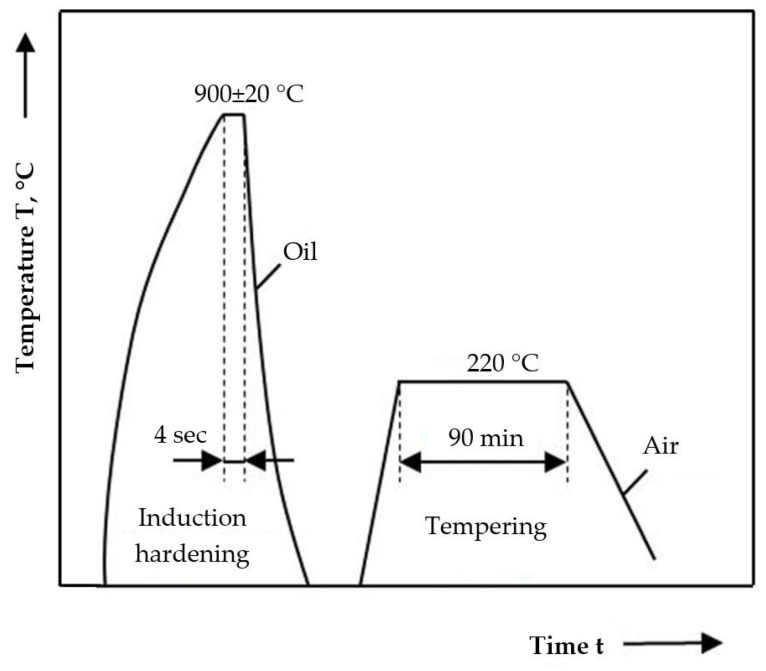
Cycle diagram of the induction hardening + low-temperature tempering heat treatment.

**Figure 4:**
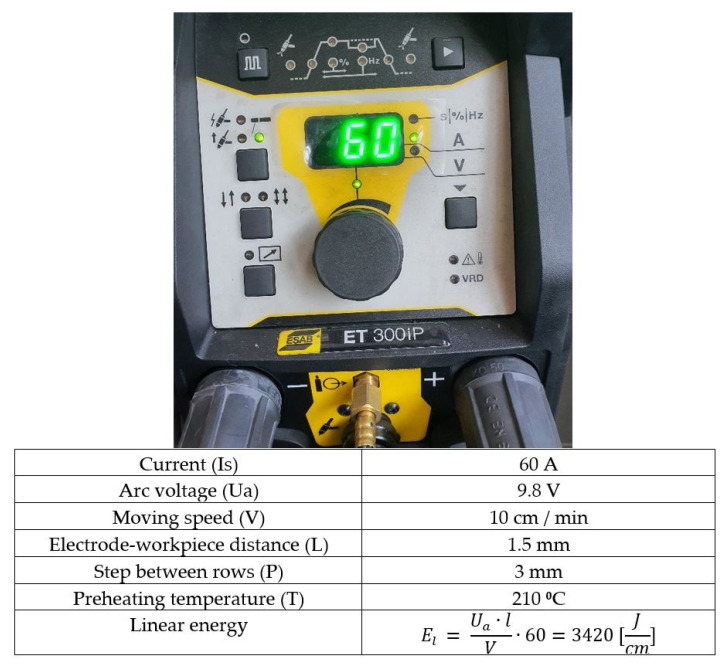
The control panel of the welding source, along with the operating parameters.

Cavitation test samples were machined from EN-GJL-200 grey cast iron and delivered as cylindrical bars with a diameter of 20 mm. To eliminate any doubt about the chemical and structural homogeneity of the material, the samples used in the experiment were processed from the same cylindrical gray cast iron bar. The samples underwent the following technological processing treatments:

- Stress relief annealing: 525 °C for 120 min followed by cooling in the furnace (Figure 2). Heating to the operating temperature was carried out at a constant, slow rate of approximately 100 °C/h to avoid the occurrence of high thermal stresses, which could overlap with the residual stresses in the parts and potentially deform or crack them. The holding time, 60 min./25 mm, is particularly necessary to achieve the desired level of stress relief. The cooling rate must be slow to prevent the introduction of thermal stresses into the already stress-relieved parts. Typically, a cooling rate of 50–60 °C/h is used.

- Induction surface hardening, followed by low-temperature tempering: 220 °C for 90 min. in the air (Figure 3); The heating of the surface layer to the austenitizing temperature is carried out at a high rate, followed by rapid cooling. This process results in a martensitic structure, leading to significant hardening only within a specific depth of the part, while the core remains unaffected by phase transformations or property changes.

- Local surface remelting using the TIG (Tungsten Inert Gas) welding technique (Figure 4); The heating current was 60 A, and the arc voltage was kept constant at 9.5–10 V. The feed rate was 10 cm/min, and the distance between the tungsten electrode (Ø 2.4 mm, L = 150 mm) and the workpiece surface was 1.5 mm. The shielding gas used was 100% argon at a flow rate of 11 L/min. The molten beads, with a width of 4 mm, overlapped by approximately 50% to ensure a uniformly treated surface. Since gray cast irons are prone to cracking, the surface remelting process was conducted under conditions of full preheating of the samples to a temperature of 210 °C.

Through experimental research [18], the optimal parameters for the TIG (Tungsten Inert Gas) local surface remelting process were defined, which significantly enhanced the resistance to cavitation erosion. These parameters are shown in Figure 4, along with an image of the control panel of the welding source.

**Figure 5:**
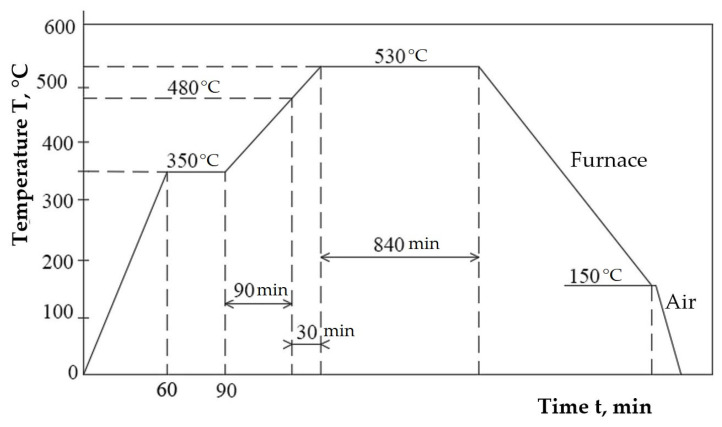
Cycle diagram of the plasma nitriding thermochemical treatment.

- Plasma nitriding: 530 °C for 840 min (Figure 5). A PROTHERM 500 system (Lebanon, TN, USA), which is intended to assist the entire process, was used as a furnace to perform plasma nitriding. According to the thermal cycle shown in Figure 5, in the first stage, the samples were preheated to 350 °C for 30 min in the retort of the system. Then, ammonia was introduced, and the heating phase continued until the process temperature of 530 °C was reached. Starting at 480 °C, ammonia dissociation occurs, releasing nitrogen atoms. After 840 min of maintaining the nitriding temperature, the samples were cooled in the treatment furnace until they reached 150 °C, at which time they were released into the atmosphere.

Cavitation erosion tests were conducted on a vibrator apparatus with piezoelectric crystals [19,20], in compliance with the requirements set by the international standards ASTM G32-2016 [21] and the laboratory’s procedure about the whole cavitation time (165 min) and the 12 intermediate periods (one of 5 min, one of 10 min, and 10 of 15 min each).

The functional parameters of the vibrator apparatus (vibration amplitude—50 μm; vibration frequency—20,000 ± 1% Hz; ultrasonic generator power—500 W; liquid medium—potable water from the public network with a temperature of 22 ± 1 °C), which determine the hydrodynamics of cavitation, specifically the destruction intensity, were rigorously controlled via a specialized software implemented in the computer connected to the vibrator apparatus [20].

Before the cavitation test, the attack surface of each sample was polished on a Buehler Phoenix Beta apparatus to a roughness of R_a_ = 0.2 ÷ 0.8 μm.

At the end of each testing period, the samples were washed under a water jet with alcohol and acetone and dried with a jet of hot air. To calculate the material loss by cavitation erosion, they were then weighed on an analytical balance of type Zat-klady Mechaniki Precyzyjnej WP 1 with a precision of five significant decimal places (up to 0.00001 grams).

A Can-on PowerShot SX200 IS camera (Tokyo, Japan) with a 12× optical zoom was used to take pictures of the samples’ surface following each testing session. This camera’s resolution also made it possible to show the level of damage throughout the entire surface. An optical microscope (Leica DM 2700 M, Wetzlar, Germany) and a scanning electron microscope (TESCAN VEGA 3 LMU, Brno, Czech Republic, Bruker EDX Quantax, Billerica, MA, USA) were used to analyze the surfaces’ morphology.

For each technological variant, sets of three samples were tested. The results of the mass losses represent the arithmetic average of the experimental determinations.

Based on the mass losses Δm_1_, Δm_2_, Δm_3_ from each time “i” for the set of 3 samples corresponding to each structural state, the mean mass loss was determined using the following formula:

(1) $$\Delta m_{i}=\sum_{j=1}^{3}\frac{\Delta m_{j}}{3}\;\mathrm{unde}\;j\;=\;1,\;2,\;3—\mathrm{sample}\;\mathrm{number}$$

The average cumulative mass loss over a specific cavitation attack duration, up to completion (165 min), was determined using the following formula:

(2) $$M_{i}=\sum_{i=1}^{12}\Delta m_{i}$$

Based on the procedure described in [19,22], the mass losses were converted into the mean cumulative depths, MDE_i_, and into the mean erosion penetration rates, MDER_i_, using the following formulas:

(3) $${\mathrm{MDE}}_{i}=\frac{4\cdot M_{i}}{\rho \cdot \pi \cdot d_{p}^{2}}[\mu m]$$

(4) $${\mathrm{MDER}}_{i}\;=\frac{{MDE}_{i}\;}{t_{i}}\;[\mu m/\min]$$

where

i—testing time;
Δm_i_—the mass of material lost due to erosion during period i, in grams;
ρ—material density, in grams/mm^3^;
Δt_i_—the duration of cavitation corresponding to period “i” (5 min, 10 min, or 15 min);
d_p_—sample surface diameter subjected to cavitation attack. (d_p_ = 15.8 mm);
ΔMDE_i_—mean erosion penetration depth value, achieved through cavitation during the period Δt_i._

## 3. Results and Discussion

### 3.1. Cavitation Curves

**Figure 6:**
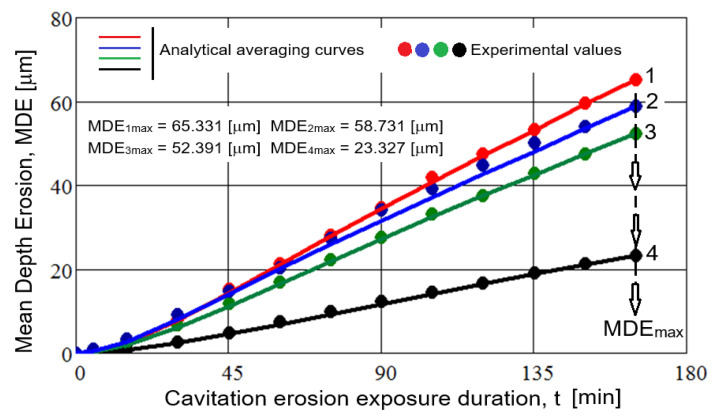
Variation in the mean erosion penetration depth with cavitation attack duration: 1—annealing for stress relief (σ = 1.659, ε ± 3%); 2—surface hardening by induction (σ = 1.035, ε ± 3%); 3—plasma nitriding (σ = 0.311, ε ± 3%); 4—local TIG remelting with I = 60 A (σ = 0.164, ε ± 3%).

**Figure 7:**
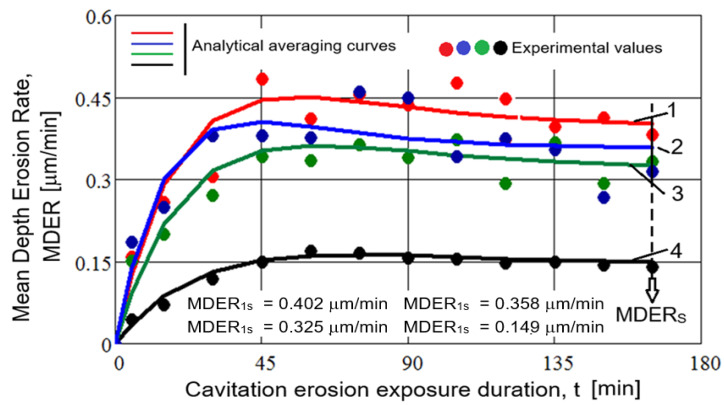
Dependence of the mean erosion penetration rate with cavitation attack duration: 1—annealing for stress relief; 2—surface hardening by induction; 3—plasma nitriding; 4—local TIG remelting with I = 60.

In Figure 6 and Figure 7, the mean experimental values of the erosion depth and erosion rate are shown, obtained from the sets of three tested samples, corresponding to different cavitation attack durations. The smoothing curves of these values, MDE(t) and MDER(t) are also presented for the four technological variants of the gray cast iron microstructure modifications considered.

To demonstrate the accuracy of the research (the reproducibility of the results from testing the three samples in each condition), the standard deviation values σ and the approximation errors ε, calculated using the equations from Ref. [20], were included in the legends of the diagrams in Figure 6.

Following the analysis of these graphs, the following observations can be made:

- The highest values, regardless of the parameter considered (mean erosion penetration depth, MDE, or mean erosion depth penetration rate, MDER), are specific to the structure obtained through the heat treatment of annealing for stress relief, while the lowest values correspond to the structure achieved by local TIG remelting (I = 60 A).
- The application of surface induction hardening, although it offers the advantage of a short processing time, results in slightly lower cavitation losses (reflected in depths and rates) compared to the annealing treatment, but still much higher than the TIG remelting technique. This phenomenon is justified by the effect of graphite, which reduces the stability of the undercooled austenite transformation, its role as a stress concentrator, and the sensitivity of the metallic matrix to stress concentration.
- Plasma nitriding results in a limited increase in cavitation resistance (higher than the structural states obtained by annealing for stress relief and surface induction hardening but lower than that obtained by TIG remelting). This phenomenon can be explained by the barrier effect of graphite in the formation of homogeneous nitrides on the alloy surface and the sensitivity of nitride particles to stress concentration. The risks of surface damage are determined by the fragility of the substrate’s chemical combination in the nitrided layer, which exfoliates during impact with shock waves and the microjets of cavitation bubbles.

According to the international standard ASTM G32-2016 [21], which sets out the methods and parameters to be used in evaluating the behavior and resistance of surfaces subjected to vibratory cavitation, the most appropriate parameter is the inverse of the mean erosion penetration rate, defined by the MDERs value (final test result, 165 min) from the MDER(t) curve, which defines cavitation resistance, R_cav_. Table 2 presents the values for this parameter for the four material processing technologies.

The data in this table show that, compared to the structural state resulting from the application of stress relief annealing, surface hardening techniques such as surface hardening by induction, plasma nitriding, and local TIG remelting lead to an increase in cavitation erosion resistance by a factor of approximately 12% to about 170%.

### 3.2. Surface Hardness Measurements

**Figure 8:**
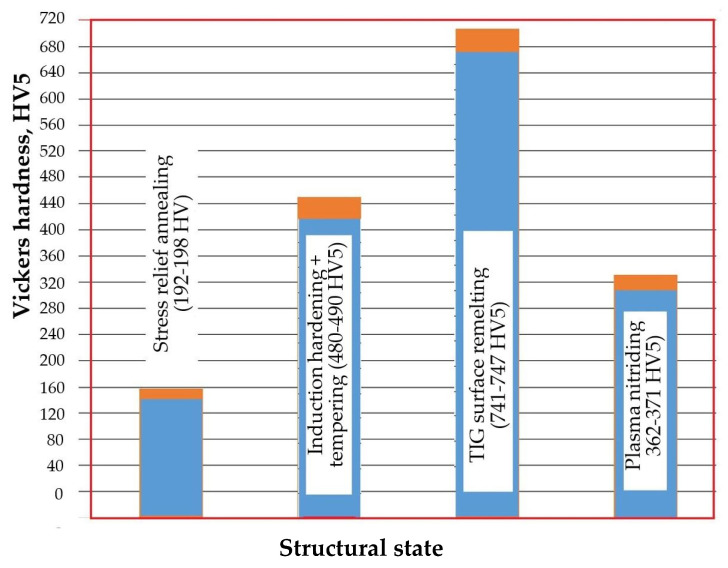
Histogram of surface hardness values [23].

It is well established that hardness is the mechanical property most sensitive to structural changes in metallic materials. On the front surface of the samples treated using the four technological variants, eight Vickers hardness measurements were taken with a 50 N load. Based on these results, the histogram presented in Figure 8 was created. The data clearly demonstrate a strong correlation between hardness and the material’s resistance to cavitation erosion. The lowest hardness values, around 195 HV5, are associated with the stress-relief annealing heat treatment, which corresponds to the highest erosion rate of approximately 0.44 µm/min. On the other hand, the local TIG remelting method of the surface using a current of I = 60 A and a linear energy of 3420 J/cm provides high hardness (approximately 744 HV5), which produces an obvious decrease in the erosion rate (to about 0.15 µm/min.). The high hardness values are attributed to the refined microstructure generated in the remelted layer.

### 3.3. Correlation of MDE—Surface Roughness

The surfaces of the samples processed using the four technological variants and tested for cavitation erosion over 165 min were subjected to roughness measurements using the Mitutoyo device. Table 3 presents the average values of the roughness parameters Ra, Rz, and Rt, which were recorded in 12–16 randomly selected areas on the cavitated surface, ranging from the periphery to the central region, along with the erosion depths (MDE) measured at the end of the cavitation test. The analysis indicates that TIG surface remelting at I = 60 A results in a significant improvement in cavitation erosion resistance, reducing the maximum MDE_max_ value by approximately 2.8 times compared to the value observed for the stress-relief annealing heat treatment. Similarly, the parameters characterizing surface roughness evolve (reductions of about 4 times for Ra, about 2.7 times for Rz, and about 3.04 times for Rt), demonstrating a good correlation with cavitation resistance.

**Figure 9:**
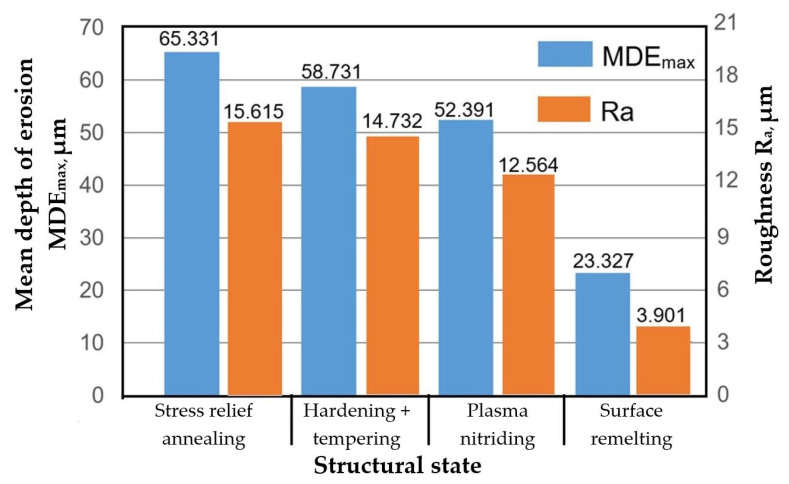
The effect of the structural state on the characteristics R_a_ and MDE_(165 min)_.

The histogram in Figure 9 clearly highlights the fact that the roughness parameter Ra is an important indicator in evaluating the cavitation erosion resistance of metallic materials and that the TIG surface remelting method for gray cast iron parts with lamellar graphite pearlitic structure brings significant benefits in increasing the service life of components operating in cavitation environments.

### 3.4. Structural Analyses

**Figure 10:**
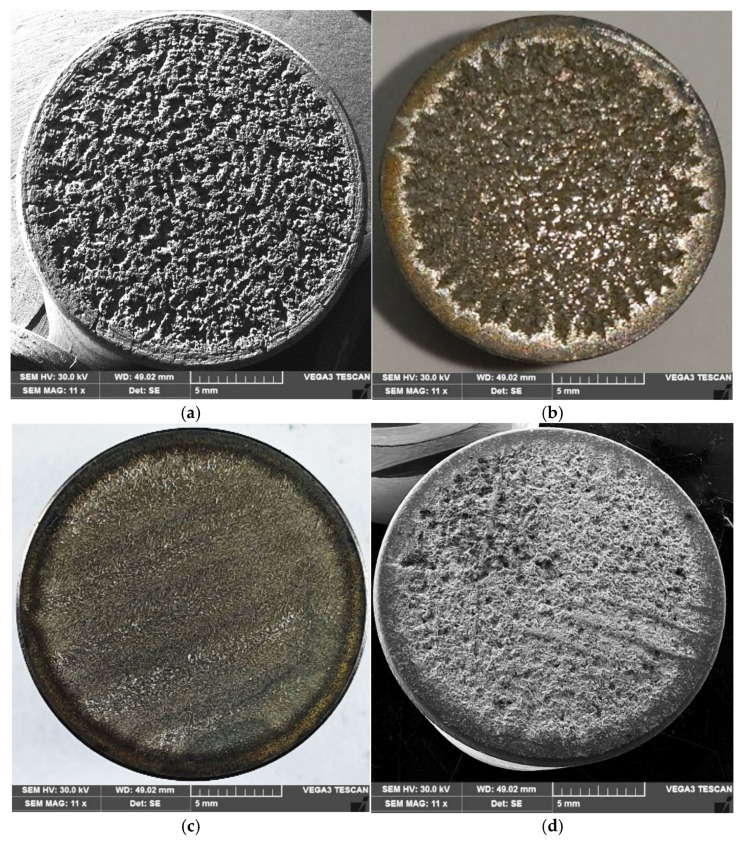
Macroscopic images of the surfaces degraded after 165 min of cavitation attack: (**a**) annealing for stress relief; (**b**) surface hardening by induction; (**c**) local TIG remelting with I = 60 A; (**d**) plasma nitriding.

In Figure 10a,b, the macroscopic images of the surfaces degraded by ultrasonic cavitation attack are presented. It can be observed that after applying the heat treatment of annealing for stress relief, the surface degradation is the most pronounced at the end of the 165 min test period (Figure 10a). In contrast, the other three processes that modify the material’s structure, resulting in progressively higher hardness, exhibit progressively less surface degradation during the cavitation erosion attack (Figure 10b–d). From this perspective, the most favorable outcome is observed with local TIG remelting.

**Figure 11:**
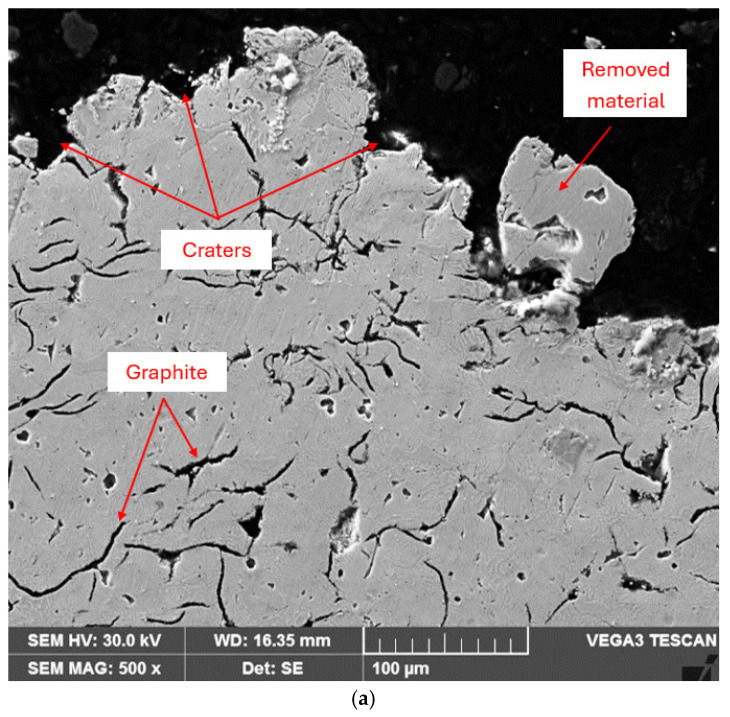

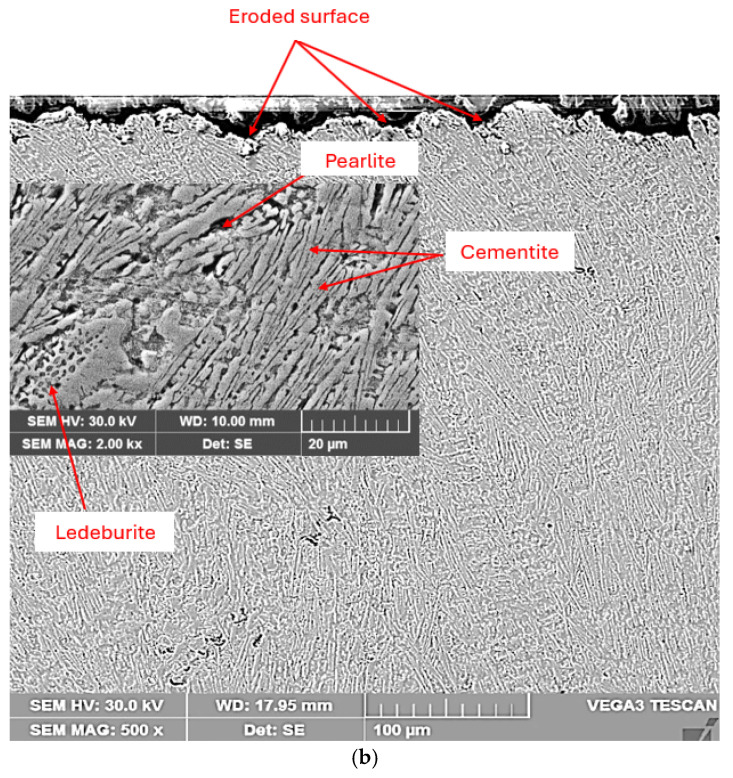
SEM micrograph (cross-sections) of the cavitated layers after 165 min: (**a**) annealing for stress relief; (**b**) local TIG remelting of the surface with I = 60 A.

Similar observations can be made from the images of cross-sections through the surface layer of samples processed by annealing for stress relief and local TIG remelting and tested for cavitation for 165 min. These images highlight significant differences in the intensity of the cavitation erosion phenomenon on the surface. Thus, the largest irregularities (Figure 11a) are identified on the surface of the material subjected to the stress relief annealing heat treatment, which results in the lowest hardness values and the highest cavitational parameters, MDE and MDER. In contrast, the surfaces hardened by local TIG remelting show the smallest surface degradation (Figure 11b).

**Figure 12:**
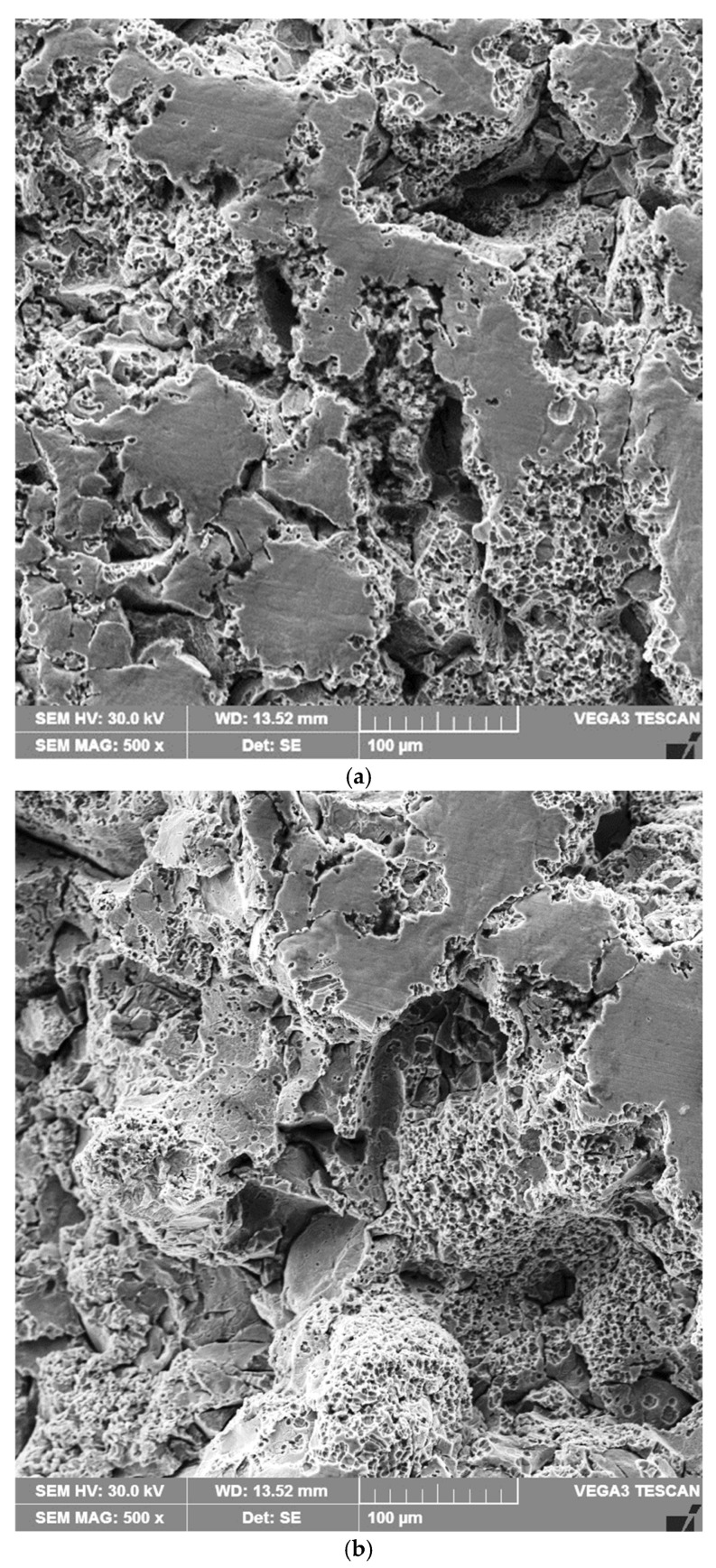

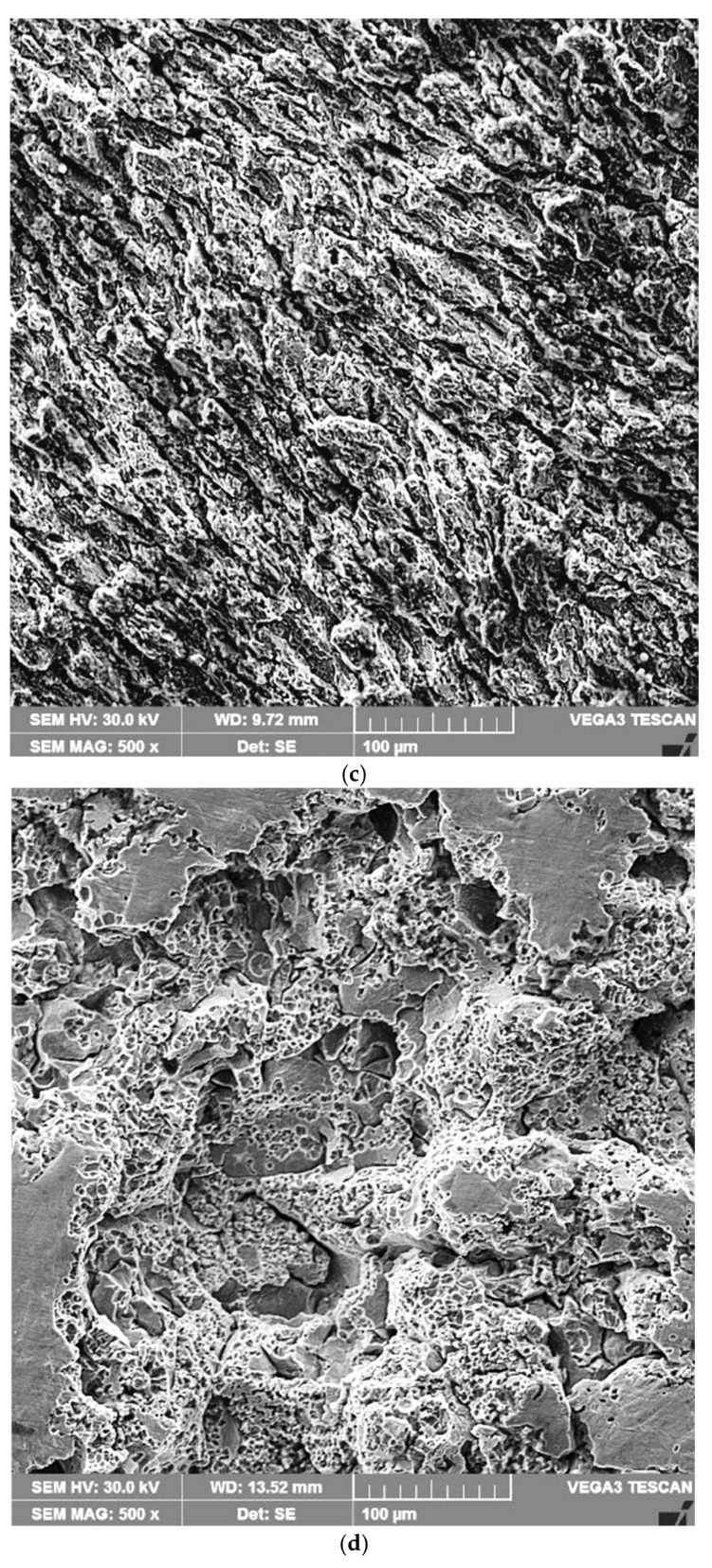
Microfractographic images of the surfaces tested for cavitation erosion for 165 min: (**a**) stress relief annealing; (**b**) surface hardening by induction followed by low-temperature tempering; (**c**) local TIG remelting of the surface; (**d**) plasma nitriding.

The microfractographic investigations performed with the scanning electron microscope on samples processed through the four technologies and subjected to cavitation tests for 165 min (Figure 12a–d) confirm the results of other research studies [10,11,14,16,24,25,26] that have demonstrated that the repeated impact exerted by microjets and shock waves on the surface causes plastic deformations, mechanical spalling, the formation of microcracks, and material loss. From the analysis of these, it can be observed that for the samples subjected to the stress relief annealing heat treatment, the intensity of the surface degradation phenomenon is maximum, manifesting as chipping in the centers of the graphite clusters, followed by fragmentation and expulsion of these. As the number of bubble collapses increases, a growth in the density of indentations along the graphite filaments, as well as pitting of the metal matrix, is observed, along with their coalescence and the formation of fatigue microcracks (Figure 12a). The topographic image of the surface of these samples (Figure 12a) highlights the formation of large-sized craters determined by the preferential cavities of the graphite lamellae, an inorganic structural constituent.

For the samples heat treated by surface induction hardening, the nucleation of fatigue cracks occurs at the separation boundaries between the graphite lamellae and the pearlitic base mass, i.e., in areas with stress concentrators. These interfaces cannot undergo mechanical spalling and, consequently, become fragile, and together with the graphite, they fragment under cavitation attack. The indentations that initially form undergo the coalescence phenomenon over time, leading to the formation of microcracks. At the end of the testing time, the attacked surface of the matrix becomes larger, with more microindentations, further indentations, and subsequent deformations of it. The removal of material due to the coalescence of these indentations led to the formation of microcraters, as observed in Figure 12b.

The physical surface modification technique through local TIG remelting manifests as a refinement of the grain and a limitation of cementite (Fe_3_C) precipitations, phenomena that increase the energy absorption capacity of the cavitation impact wave, delaying the nucleation of cracks. In comparison with the surface morphology characteristic of the stress relief annealing heat treatment, the degradation process is much less intense and much more uniform (Figure 12c).

For plasma nitriding samples, the surface morphology at the end of the cavitation tests does not significantly differ from that characteristic of the surface induction hardening treatment. Although the marginal layer has a relatively high hardness, the eroded surface shows the formation of deep craters in the places where former fragile nitride particles were present in the chemical combination subzone (Figure 12d).

## 4. Conclusions

Following the structural investigations and analysis of the cavitation erosion characteristics of gray cast irons with a pearlitic matrix and lamellar graphite, the following conclusions can be drawn:

- Compared to the stress relief annealing heat treatment, applying either surface hardening by induction or plasma nitriding results in only a 12% and 24% increase in cavitation erosion resistance, respectively.
- The technique of local TIG remelting of the surface, operated at a current of 60 A and a linear energy of 3420 J/cm, provides a significant enhancement in cavitation erosion resistance, with a factor of approximately 170%.
- low hardness values (approximately 195 HV5), characteristic for stress relief annealing treatment, contribute to the highest cavitation erosion rates (approximately 0.44 µm/min), while high hardness values (approximately 744 HV5), specific to local TIG remelting, favor a pronounced reduction in erosion rate (approximately 0.15 µm/min).
- The removal of graphite flakes through local TIG remelting, resulting in a refined and homogeneous microstructure with high hardness, undergoes slower and more uniform degradation, with extremely fine pitting.

## Figures and Tables

**Table 1:** EN-GJL-200 grey cast iron chemical composition, wt.%.

Carbon (C)	3.26%
Silicon (Si)	1.94%
Manganese (Mn)	0.90%
Sulfur (S)	0.11%
Phosphorous (P)	0.12%
Iron (Fe)	Balance

**Table 2:** The effect of hot processing processes on the characteristics of MDER_s_ and R_cav._

Structural State	The Parameter of Cavitation Erosion Resistance		Variation Compared to Stress Relief Annealing
	MDERs [µm/min]	Rcav. [min/µm]	
Stress relief annealing	0.402	2.487	-
Induction hardening followed by low-temperature tempering	0.358	2.793	increase by about 12%
Plasma nitriding	0.325	3.076	increase by about 24%
TIG local remelting	0.149	6.711	increase by about 170%

**Table 3:** Comparative values of roughness parameters and erosion depths MDE_max_.

Sample State	MDE_max_ [µm]	Ra [µm]	Rz [µm]	Rt [µm]
Stress relief annealing	65.331	15.615	61.583	78.581
Surface hardening by induction followed by low-temperature tempering	58.731	14.732	58.963	62.621
Plasma nitriding	23.327	3.901	22.739	25.842
TIG local remelting	52.391	12.564	56.344	60.488
Processing effects (reference state: stress relief)				
Processing effects (reference state: Induction hardening + low tempering)				
TIG remelting with I = 60 A	Decrease with 2.5 times	Decrease with 3.8 times	Decrease with 2.6 times	Decrease with 2.4 times
Plasma nitriding	Decrease with 12%	Decrease with 17%	Decrease with 4.6%	Decrease with 3.5%

## Data Availability

The original contributions presented in this study are included in the article. Further inquiries can be directed to the corresponding author.
